# The Prevalence, Risk Factors, and Antimicrobial Resistance Determinants of *Helicobacter pylori* Detected in Dyspeptic Patients in North–Central Bangladesh

**DOI:** 10.3390/idr16020014

**Published:** 2024-02-22

**Authors:** Syeda Jannatul Ferdaus, Shyamal Kumar Paul, Syeda Anjuman Nasreen, Nazia Haque, Mohammad Sadekuzzaman, Mohammad Reazul Karim, Syed Mahmudul Islam, Abdullah Al Mamun, Fardousi Akter Sathi, Proma Basak, Rifat Binte Nahid, Suraiya Aktar, Nobumichi Kobayashi

**Affiliations:** 1 Department of Microbiology, Mymensingh Medical College, Mymensingh 2200, Bangladesh; jannat33bcs@gmail.com (S.J.F.); nasreenm19@gmail.com (S.A.N.); drnaziahaque@gmail.com (N.H.); aamamun6985@gmail.com (A.A.M.); drsathi.dmc@gmail.com (F.A.S.); nahid.r109@gmail.com (R.B.N.); 2 Netrokona Medical College, Netrokona 2400, Bangladesh; drshyamal10@yahoo.com; 3 Department of Livestock Services, Central Disease Investigation Laboratory (CDIL), 48, KaziAlauddin Road, Dhaka 1000, Bangladesh; numanmicro@gmail.com; 4 Department of Gastroenterology, Mymensingh Medical College, Mymensingh 2200, Bangladesh; reazul1975@gmail.com; 5 Department of Microbiology and Hygiene, Faculty of Veterinary Science, Bangladesh Agricultural University, Mymensingh 2202, Bangladesh; mahmudulislamrana1@gmail.com; 6 Shaheed Syed Nazrul Islam Medical College, Kishoreganj 2300, Bangladesh; dr.promabasak@gmail.com; 7 Department of Microbiology, Dhaka Central International Medical College and Hospital, 2/1 Ring Road, Shyamoli, Dhaka 1207, Bangladesh; suraiyaaktar750@gmail.com; 8 Department of Hygiene, School of Medicine, Sapporo Medical University, S-1 W-17, Chuo-ku, Sapporo 060-8556, Japan

**Keywords:** *Helicobacter pylori*, dyspeptic patients, Bangladesh, antimicrobial resistance, 23S rRNA, PBP-1A, *rdxA*

## Abstract

Chronic infection of *Helicobacter pylori* represents a key factor in the etiology of gastrointestinal diseases, with high endemicity in South Asia. The present study aimed to determine the prevalence of *H. pylori* among dyspeptic patients in north–central Bangladesh (Mymensingh) and analyze risk factors of infection and antimicrobial resistance (AMR) determinants in the pathogen. Endoscopic gastrointestinal biopsy samples were collected from dyspeptic patients for a one-year period from March 2022 and were checked for the presence of *H. pylori* via the rapid urease test and PCR and further analyzed for the status of virulence factors *vacA*/*cagA* and genetic determinants related to AMR via PCR with direct sequencing or RFLP. Among a total of 221 samples collected, 80 (36%) were positive for *H. pylori*, with the *vacA*+/*cagA*+ genotype being detected in almost half of them. *H. pylori* was most prevalent in the age group of 41–50-year-olds, with it being more common in males and rural residents with a lower economic status and using nonfiltered water, though the rates of these factors were not significantly different from those of the *H. pylori*-negative group. Relatively higher frequency was noted for the A2147G mutation in 23S rRNA, related to clarithromycin resistance (18%, 7/39). Amino acid substitutions in PBP-1A (T556S) and GyrA (N87K and D91N) and a 200 bp deletion in *rdxA* were detected in samples from some patients with recurrence after treatment with amoxicillin, levofloxacin, and metronidazole, respectively. The present study describes the epidemiological features of *H. pylori* infection in the area outside the capital in Bangladesh, revealing the spread of AMR-associated mutations.

## 1. Introduction

*Helicobacter pylori* is one of the most common pathogenic bacteria in humans and is associated with the pathogenesis of gastritis, gastroduodenal ulcers, gastric cancer, and other diseases worldwide [1]. This bacterium colonizes the epithelium of the human stomach, and its prevalence is typically 80–90% in developing countries, which is higher than in developed countries (<40%) [2]. Among the *H. pylori*-positive population, 10–20% are estimated to have a lifetime risk of developing ulcerative disease, while 1–2% have a risk of gastric cancer [3]. It has become evident from a number of clinical trials that *H. pylori* eradication therapy is beneficial to symptomatic patients by preventing the progression of gastric diseases. Although the regimens of eradication therapy have been designed and widely applied, successful eradication of *H. pylori* is still a global challenge. The increased risk of *H. pylori* infection is related to environmental factors, including living in low-income countries and having a lower socioeconomic status [4], occupation, water supply, and dietary habits [5,6]. Accordingly, to clarify such risk factors may contribute to the prevention of *H. pylori*-associated diseases.

In South Asia, higher seroprevalence of *H. pylori* in asymptomatic populations was described (>90% in Bangladesh; ~81% in India) compared with other Asian regions more than two decades ago [7,8]. In a recent epidemiological study of *H. pylori* in Bangladesh, its prevalence was reported to be 33–47% in gastroduodenal biopsy samples obtained through the rapid urease test (RUT) and PCR [9,10]. For the eradication of *H. pylori*, a triple therapy including a proton pump inhibitor combined with two antimicrobials, amoxicillin (AMX) and clarithromycin (CLA) or metronidazole (MNZ), is the standard first-line regimen. In Bangladesh, a CLA-based triple regimen remains the first option, and levofloxacin (LVX)-based triple therapy is recommended as a second option after failure of CLA-containing therapy [11]. However, high rates of resistance of *H. pylori* to CLA (10–30%), MNZ (78–95%), and LVX (66%) have been documented in some reports, in contrast to a low rate against AMX (3.6–6.6%) [12,13,14]. Furthermore, in Bangladesh, mutations responsible for antimicrobial resistance (AMR) were detected in *H. pylori* genes, including 23S rRNA, *gyrA*, and *rdxA* [15,16]. The reported high rate of AMR associated with the presumptive high prevalence of *H. pylori* provides a rationale for its monitoring in the country. Nevertheless, the available information on the prevalence and AMR of *H. pylori* were obtained mostly in the capital city, Dhaka, and based on somewhat older study subjects, indicating the need for updated data from a region other than the capital to understand the present situation of *H. pylori* infection in this country. In the present study, we examined the latest prevalence of *H. pylori* in dyspeptic patients in an area distant from Dhaka and risk factors and AMR with its genetic determinants.

## 2. Materials and Methods

This study was conducted as a cross-sectional, observational study. Endoscopic gastroduodenal biopsy specimens were collected from dyspeptic patients attending the gastroenterology department of Mymensingh Medical College Hospital. Patients who presented with symptoms of dyspepsia for more than one month were included in this study, and dyspeptic symptoms were defined as one or more of the following: (1) upper abdominal or lower chest pain with or without food intake, (2) regurgitation, heartburn, and water brash, (3) anorexia, nausea, and vomiting, and (4) bloating, belching, and flatulence. The exclusion criteria were as follows: (1) individuals over 65 years of age and those who had a severe medical or surgical illness such as asthma, chronic obstructive pulmonary disease, previous gastric surgery, etc., (2) medication history of proton pump inhibitors, nonsteroidal anti-inflammatory drugs, colloidal bismuth compounds, or antibiotics for the eradication of *H. pylori* over the past four weeks, and (3) previously diagnosed stomach cancer. From one patient, only one specimen was used for this study. The specimens were stored at −80 °C until they were analyzed. The patients’ information, including their clinical history, lifestyle habits, and risk factors, was recorded in the case report form via interview.

Detection of *H. pylori* in the specimens was performed via RUT using Christensen’s urea agar media (HiMedia laboratories pvt Ltd., Mumbai, India) and PCR targeting 16S rRNA and *ureC* (*glmM*) with primers and conditions as described previously [17,18,19]. For *H. pylori* PCR-positive samples, the presence of *vacA* and *cagA* was examined via PCR as described previously [20,21]. In the PCR of *vacA*, genotypes s1 and s2 were discriminated by product size with primers VA1-F and VA1-R [20]. In this study, *H. pylori*-positive patients who received antimicrobials for *H. pylori* eradication therapy 6–12 months previous were regarded as those having inappropriate therapy or carrying antimicrobial-resistant *H. pylori* strains. Therefore, samples from these patients were selected for analysis of the presence of genetic mutations related to AMR. Partial nucleotide sequences of the PBP-1A gene and *gyrA* were determined via Sanger sequencing of PCR products to detect mutations that are responsible for resistance to AMX and LVX, respectively [22,23]. Deletion of the *rdxA* gene, one of the genetic mechanisms of MNZ resistance, was examined via PCR to identify the different sizes of PCR products [24]. A2147G mutations (formerly described as A2143G [25]) in 23S rRNA, a common mechanism of CLA resistance, were analyzed by PCR-RFLP as described previously [24]. To confirm species of *H. pylori*, the sequences of partial 16S rRNA gene, 23srRNA, *cagA*, and *ureC* were determined for representative samples (JHP_100, JHP_103, JHP_101, and JHP_102) and deposited to GenBank under accession numbers OQ247937, OQ379921, OQ319145, and OQ319146, respectively.

The difference in the rate of risk factors and variables of patients between *H. pylori*-positive and -negative patients was statistically analyzed via Fisher’s exact test with the use of js-STAR XR ver.1.1.9 software (https://www.kisnet.or.jp/nappa/software/star/index.htm (accessed on 22 December 2023)). A *p*-value < 0.05 was considered statistically significant.

## 3. Results

During the study period, a total of 221 gastroduodenal specimens were collected from patients with dyspeptic symptoms. Of them, 72 and 71 samples were found to be positive for *H. pylori* via RUT and PCR targeting the 16S rRNA gene as well as *ureC*, respectively, and 63 samples were found to be positive via both screening methods. In the present study, *H. pylori*-positive cases were defined as those positive in either the RUT or PCR. Accordingly, 80 cases (36% of all of the study subjects) were determined as having *H. pylori* infection. The most common endoscopic finding in *H. pylori*-positive cases was antral gastritis, followed by erosive gastritis and nodular gastritis (Table 1). However, between *H. pylori*-positive and -negative cases, no significant difference was found in the proportion of clinical findings. In duodenal ulcers, PCR showed a slightly higher detection rate of *H. pylori* than the RUT.

*H. pylori* was most prevalent in the age group of 41–50-year-olds (40%), followed by 31–40-year-olds (31%) and 21–30-year-olds (18%), with it being more common in males (61%) than females (39%) (Table 2). Among the *H. pylori*-positive cases (*n* = 80), 74% (*n* = 59) lived in rural areas, with 63% (*n* = 50) having a lower socioeconomic status. Rates of smoking, use of nonfiltered water, and a family history of peptic ulcer disease (PUD) were higher in *H. pylori*-positive cases than in *H. pylori*-negative cases. However, differences in the prevalence of the demographic variables and risk factors analyzed were not statistically significant between the *H. pylori*-positive and -negative groups.

Among the 44 *H. pylori*-positive samples which were available for PCR targeting virulence factor genes, 36 were positive for both *vacA* and *cagA*, while 8 were *vacA*(+)/*cagA*(−) (Table 3). Among the 44 *vacA*-positive samples, most of them showed the s1 genotype (*n* = 43), while only one sample showed the s2 genotype. *vacA*(+)/*cagA*(+) was found in 67–86% of cases of PUD, gastritis, and gastroesophageal reflux disease (GERD).

Major mutations related to resistance to CLA, MNZ, LVX, and AMX were analyzed for selected cases that had undergone antimicrobial therapy 6–12 months prior, with approximately 39 (49%), 30 (38%), 5 (6%), and 5 (6%) *H. pylori*-positive cases, respectively (Table 4). A2147G substitution causing CLA resistance was identified via PCR-RFLP (Figure S1a). This mutation was found in 18% (7/39) of samples tested. A 200-bp deletion in *rdxA* was detected in only two samples (7%) via PCR to discriminate different sizes of product (Figure S1b). In PBP-1A, the T556S substitution that alters the penicillin-binding motif (KTG to KSG) was detected in one specimen. Among the five LVX-resistant cases, two different substitutions in the quinolone resistance determining region (QRDR) were detected in three samples from three cases.

## 4. Discussion

*H. pylori* infection is recognized as being involved in a subset of dyspepsia, which represents a wide spectrum of gastrointestinal disorders affecting up to 25% of the population sporadically [26]. In Bangladesh, as well as other South Asian countries, high seroprevalence of *H. pylori* has been described since the 1990s [8], with 91% being seropositive in the young healthy male population [7]. Among children in a poor peri-urban community, prevalence measured via the urea breath test was reported to be 68% while varying depending on age group [27]. While there was a dearth of information in the 2000s, a histological study on the gastric mucosa of patients with abdominal complaints revealed the infection rate of *H. pylori* to be 60.2% (2008–2010) [28]. More recent studies on adult dyspeptic patients reported the overall *H. pylori* prevalence to be 47% (44% with the RUT) (2015) [9] and 32.9% (with the RUT, PCR, and histology) (2018–2019) [10]. These detection rates of *H. pylori* were comparable to our present study showing a prevalence of 36% (the RUT and PCR) among dyspeptic patients in north–central Bangladesh (Mymensingh). Therefore, the recent prevalence of *H. pylori* among dyspeptic patients in the country seems to be similar. In other Asian and African countries, a similar prevalence of *H. pylori* was described, e.g., 40.9% (the CLO test) in Qatar [29], 36% (histology) in Uganda [30], and 31.2% (histology, the RUT, and culture) in Iran [31]. Nevertheless, the notably high detection rate of *H. pylori* (PCR and the CLO test) (67%) was shown in a study in southern Bangladesh (Chittagong) [32], suggesting the presence of highly endemic areas within the country. Although recent information on seroprevalence is limited, Sarker and coworkers reported 86.8% and 67.5% seropositivity in gastric cancer patients and controls, respectively (2013–2014), in Dhaka [33], indicating that high prevalence was maintained in some populations. Globally, the prevalence of *H. pylori* is considered to be decreasing continuously [6]. With the spread of eradication therapy for *H. pylori* infection and the improvement of hygienic conditions, a reduction in *H. pylori* prevalence is expected in Bangladesh. To confirm the change in prevalence, continuous monitoring of the same area/population with the same detection method for *H. pylori* may be necessary.

In the present study, the *H. pylori*-positive rate was higher in males and in the 41–50-year age group, which was similar to that observed in another study in Bangladesh [10]. Although a significant difference was not found, *H. pylori*-positive cases showed a higher proportion of some risk factors, e.g., residence in rural areas, nonfiltered water, and smoking, compared with *H. pylori*-negative cases. This finding is consistent with the view that *H. pylori* infection is associated with dietary habits and lifestyle, as described previously [5,6]. However, >60% of both *H. pylori*-positive and -negative cases were categorized into a group of lower socioeconomic status in our study, probably due to local areas in low-income country. This finding suggests that dyspepsia in the study population was also related to factors or pathogens other than *H. pylori*, which might be linked to lower socioeconomic status and low hygienic conditions.

According to a systematic review of AMR in *H. pylori* in the Asia–Pacific region, the highest resistance rate was described against MNZ (61%), followed by LVX (35%), CLA (30%), and AMX (6%), and there was an increasing trend in the resistance rates [34]. The prevalence of AMR was shown to be remarkable in South Asian countries [35]. In Bangladesh, high resistance rates to MNZ (96%), LVX (66%), and CLA (39%) were reported for isolates in 2014 [13,14], though the resistance rates to MNZ and CLA in 1999–2001 were lower (77.5% and 10%, respectively) [12]. In the present study, though the exact AMR rate was not evaluated, patients who might have inappropriate therapy or AMR strains were more frequent for those who received CLA and MNZ previously (49% and 38% of *H. pylori*-positive patients, respectively) than LVX and AMX. These findings contrast with the previously published results in Bangladesh [13,14] and suggest the increasing trend of resistance to CLA and a relative decrease in MNZ resistance. Such a difference in the rate of AMR may be related to the practice of eradication therapy in our study site.

Genetic mechanisms causing resistance to CLA, LVX, and MNZ in *H. pylori* detected in our present study were also described in recent studies in Bangladesh [15,16], indicating the potential spread of these resistance mechanisms. The A2147G mutation in 23S rRNA detected in 18% of specimens in our study was also reported in 45.5% of CLA-resistant strains [16], showing its dominance as a resistance mechanism. N87K and D91N in GyrA detected in our study are also described as the most frequent mutations in LVX-resistant strains [16]. In contrast, T556S in PBP-1A detected in our study has never been found in Bangladesh previously, though other mutations in PBP-1a, -2, -3, and -4 have been reported [16]. The T556S substitution is located in the penicillin-binding motif and was demonstrated to confer AMX resistance [36] and found in clinical isolates in Argentina [37,38], while this mutation has been rarely reported. Identification of this PBP-1A mutation in Bangladesh suggests the spread of AMX resistance, which may indicate the need for prudent eradication therapy for *H. pylori* infection.

This study has some limitations. For the detection of *H. pylori*, only one biopsy sample was taken from a patient, which might cause false-negative results because of the irregular distribution of bacteria in the gastric mucosa, resulting in a potentially lower positive rate. Thus, it is possible that the significant difference in incidence rates of any risk factors might not be evident between *H. pylori*-positive and -negative groups. In the analysis of the genetic mechanisms of AMR, only a limited number of genes and mutations were examined. Although the targeted mutations were detected, the presence and frequency of other genetic mechanisms were undetermined.

The present study revealed the prevalence of *H. pylori* in dyspeptic patients in north-central Bangladesh, along with the risk factors, AMR, and a part of their genetic mechanisms. Though the prevalence of *H. pylori* was comparable to that in the capital city, Dhaka, considering the presence of potential false-positive samples, the actual prevalence is suggested to be higher. Therefore, further surveillance of *H. pylori* via accurate diagnostic methods is required, particularly in various rural areas within the country. In addition, periodic studies in the same location will also be valuable to describe the trend of prevalence and risk factors for *H. pylori* infection. Regarding AMR, the surveillance of resistance rates to CLA and MNZ may be primarily important. The genetic mechanisms of resistance should be thoroughly clarified since the available information is still limited in Bangladesh. The diversity and frequency of the resistance mechanisms may reveal a potential spread of specific resistant strains, which will provide basic information to contribute to the control of *H. pylori* infection.

## Figures and Tables

**Table 1:** Clinical outcome of patients in cases with and without *H. pylori* infection.

Endoscopic Finding/Disease Type (Total *n* = 221)	Number of *H. pylori*-Positive Samples (% in Endoscopic Finding)		Number of Cases (% in Cases)		
	RUT (*n* = 72)	PCR (*n* = 71)	*H. pylori*-Positive (*n* = 80 *^2^)	*H. pylori*-Negative (*n* = 141)	*p*-Value
Normal mucosa (6)	1 (17%)	1 (17%)	1 (1.3%)	5 (3.5%)	0.421
Antral gastritis (52)	19 (37%)	18 (35%)	22 (28%)	30 (21%)	0.324
Erosive gastritis (50)	17 (34%)	16 (32%)	17 (21%)	33 (23%)	0.741
Nodular gastritis (35)	12 (34%)	13(37%)	13 (16%)	22 (16%)	1.000
Gastric ulcer (29)	7 (24%)	5 (17%)	7 (8.8%)	22 (16%)	0.103
Duodenal ulcer (31)	8 (26%)	12 (39%)	12 (15%)	19 (13%)	0.559
Gastric cancer (3)	1 (33%)	1 (33%)	1 (1.3%)	2 (1.4%)	1.000
GERD *^1^ (15)	7 (47%)	5 (33%)	7 (8.8%)	8 (5.7%)	0.412

*^1^ gastroesophageal reflux disease. *^2^ *H. pylori*-positive cases by RUT and/or PCR.

**Table 2:** Demographic and risk factors of *H. pylori*-positive and -negative cases.

Variable	Number of Cases (%)		
	*H. pylori*-Positive (*n* = 80)	*H. pylori*-Negative (*n* = 141)	*p*-Value
Age group (years)			
<20 (18–20)	2 (2.5%)	8 (5.6%)	0.335
21–30	14 (18%)	31 (22%)	0.489
31–40	25 (31%)	38 (27%)	0.537
41–50	32 (40%)	47 (33%)	0.248
51–60	6 (7.5%)	14 (10%)	0.632
61–65	1 (1.2%)	3 (2.1%)	1.000
Sex: male/female	49 (61%)/31 (39%)	82 (58%)/59 (42%)	0.672
Residential area			
Rural	59 (74%)	93 (66%)	0.290
Urban	21 (26%)	48 (34%)	0.290
Risk factors			
Lower socioeconomic status *^1^	50 (63%)	102 (73%)	0.134
Consumption of restaurant food, meat	10 (12.5%)	30 (27%)	0.145
Nonfiltered water	48 (60%)	70 (50%)	0.161
Family history of PUD	30 (38%)	45 (32%)	0.460
Smoking	37 (46.2%)	50 (35%)	0.538

*^1^ Estimated annual income of patient was used to classify this category based on criteria of the World Bank Atlas Method (low-income economics, July 2021).

**Table 3:** Virulence genes and endoscopic finding of *H. pylori* (*n* = 44).

Virulence Gene Status	No. of Samples	No. of Cases with Clinical Finding (% in the Finding)			
		PUD (*n* = 11)	Gastritis (*n* = 29)	GERD (*n* = 3)	Others (*n* = 1)
*vacA*+, *cagA+*	36	8 (73)	25 (86)	2 (67)	1 (100)
*vacA*+*, cagA*−	8	3 (27)	4 (14)	1 (33)	0

**Table 4:** Mutations related to antimicrobial resistance in *H. pylori*.

Antimicrobial Resistance Observed Clinically	Gene Analyzed	Mutation Identified	No. of Samples with Resistance Suspected by Clinical Course (% in 80 *H. pylori*-Positive Samples)	No. of Samples with Mutation (% in Examined Samples)
Clarithromycin	23S rRNA	A2147G	39 (49%)	7 (18%)
Metronidazole	*rdxA*	200-bp deletion	30 (38%)	2 (7%)
Amoxicillin	PBP-1A	T556S	5 (6%)	1 (20%)
Levofloxacin	GyrA	N87K	5 (6%)	1 (20%)
		D91N	5 (6%)	2 (40%)

## Data Availability

In this manuscript, additional data is only Supplementary Material Figure S1.

## Supplementary Materials

The following supporting information can be downloaded at: https://www.mdpi.com/article/10.3390/idr16020014/s1. Figure S1: Detection of mutations in 23S rRNA and *rdxA* associated with resistance to clarithromycin and metronidazole, respectively.
