# Supplementary material for: The Prevalence, Risk Factors, and Antimicrobial Resistance Determinants of Helicobacter pylori Detected in Dyspeptic Patients in North–Central Bangladesh
Source: Infect Dis Rep. 2024 Feb 22;16(2):181–8. doi: 10.3390/idr16020014 (PMC10961801; doi:10.3390/idr16020014)
Supplement: Supplementary file 1 [file idr-16-00014-s001.zip › idr-2847771-supplementary.pptx]

## Slide 1
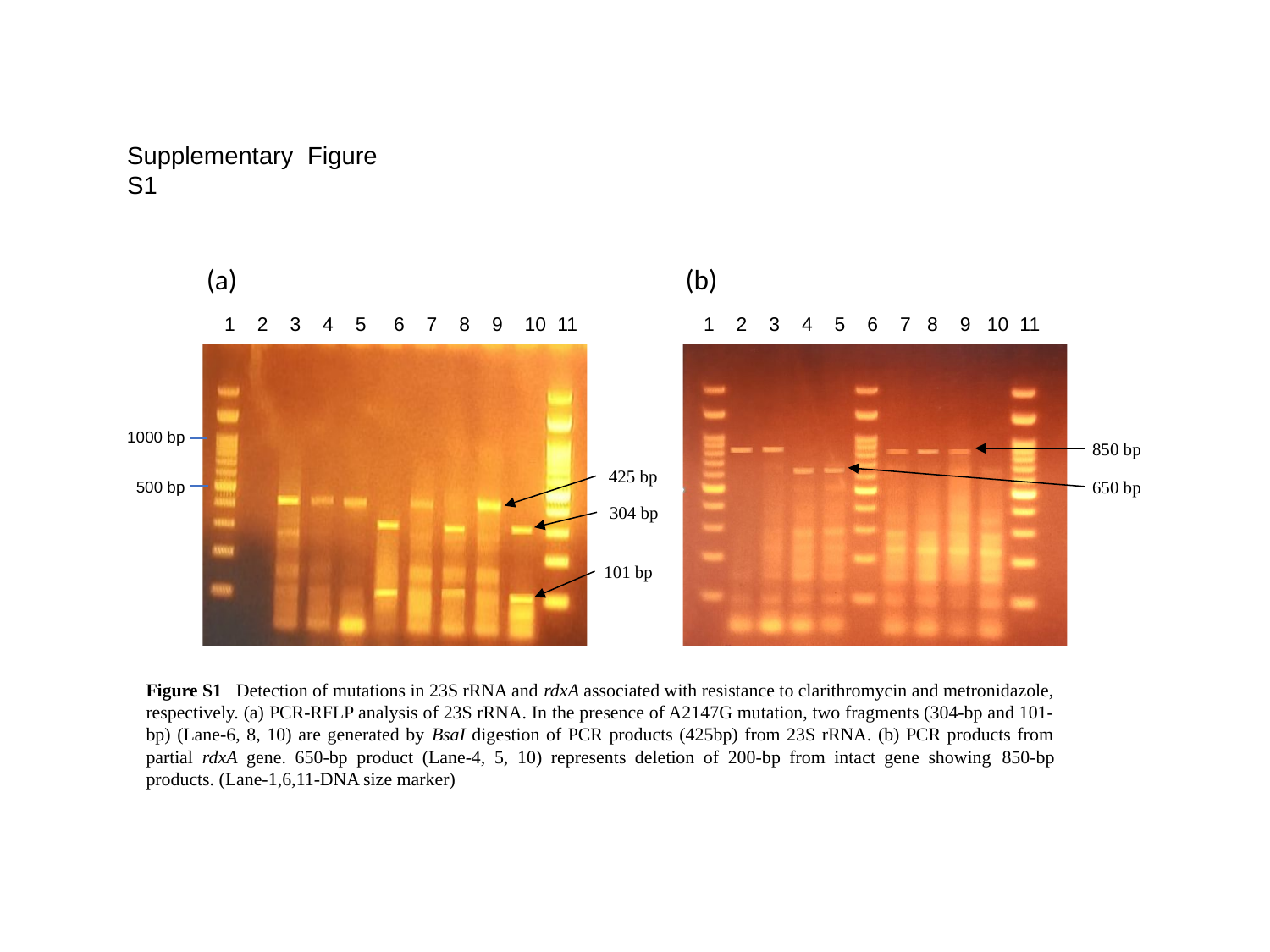

Supplementary Figure S1
(a)
(b)
1 2 3 4 5 6 7 8 9 10 11
1 2 3 4 5 6 7 8 9 10 11
1000 bp
850 bp
425 bp
650 bp
500 bp
304 bp
101 bp
Figure S1 Detection of mutations in 23S rRNA and rdxA associated with resistance to clarithromycin and metronidazole, respectively. (a) PCR-RFLP analysis of 23S rRNA. In the presence of A2147G mutation, two fragments (304-bp and 101-bp) (Lane-6, 8, 10) are generated by BsaI digestion of PCR products (425bp) from 23S rRNA. (b) PCR products from partial rdxA gene. 650-bp product (Lane-4, 5, 10) represents deletion of 200-bp from intact gene showing 850-bp products. (Lane-1,6,11-DNA size marker)
